# Astroglial Connexin 43 Regulates Synaptic Vesicle Release at Hippocampal Synapses

**DOI:** 10.3390/cells12081133

**Published:** 2023-04-11

**Authors:** Giselle Cheung, Oana Chever, Astrid Rollenhagen, Nicole Quenech’du, Pascal Ezan, Joachim H. R. Lübke, Nathalie Rouach

**Affiliations:** 1 Neuroglial Interactions in Cerebral Physiology and Pathologies, Center for Interdisciplinary Research in Biology, Collège de France, CNRS, INSERM, Labex Memolife, Université PSL, 75231 Paris, France; 2 Institute for Neuroscience and Medicine INM-10, Research Center Jülich, 52428 Jülich, Germany; 3 Jülich-Aachen Research Alliance Translational Brain Medicine, 52056 Aachen, Germany; 4 Department of Psychiatry, Psychotherapy and Psychosomatics, Rheinisch-Westfaelische Technische Hochschule Aachen University, 52056 Aachen, Germany

**Keywords:** astrocytes, connexin 43, synaptic vesicle, release probability, synaptic transmission, hippocampus

## Abstract

Connexin 43, an astroglial gap junction protein, is enriched in perisynaptic astroglial processes and plays major roles in synaptic transmission. We have previously found that astroglial Cx43 controls synaptic glutamate levels and allows for activity-dependent glutamine release to sustain physiological synaptic transmissions and cognitiogns. However, whether Cx43 is important for the release of synaptic vesicles, which is a critical component of synaptic efficacy, remains unanswered. Here, using transgenic mice with a glial conditional knockout of Cx43 (Cx43−/−), we investigate whether and how astrocytes regulate the release of synaptic vesicles from hippocampal synapses. We report that CA1 pyramidal neurons and their synapses develop normally in the absence of astroglial Cx43. However, a significant impairment in synaptic vesicle distribution and release dynamics were observed. In particular, the FM1-43 assays performed using two-photon live imaging and combined with multi-electrode array stimulation in acute hippocampal slices, revealed a slower rate of synaptic vesicle release in Cx43−/− mice. Furthermore, paired-pulse recordings showed that synaptic vesicle release probability was also reduced and is dependent on glutamine supply via Cx43 hemichannel (HC). Taken together, we have uncovered a role for Cx43 in regulating presynaptic functions by controlling the rate and probability of synaptic vesicle release. Our findings further highlight the significance of astroglial Cx43 in synaptic transmission and efficacy.

## 1. Introduction

Astrocytes are known for their diverse roles in synaptic transmission, which is largely facilitated by the close physical interactions between their fine perisynaptic astroglial processes (PAPs) and the pre- and post-synaptic compartments together forming the “tripartite synapse” [1,2,3]. One of their many important functions is the removal of excess glutamate and potassium upon synaptic activity, such that continuous neuronal communications can be sustained while preventing the saturation of postsynaptic neurotransmitter receptors [4,5,6]. In the hippocampus, astrocytes regulate the heterogeneity of presynaptic strength [7]. Another crucial role of astrocytes is to replenish released glutamate in presynaptic compartments by providing a constant supply of glutamine, which is then converted back to glutamate upon uptake by neurons during the process that is called the glutamate–glutamine cycle [8]. We are, however, only just beginning to understand the intricate details and potential molecular mechanisms mediating astrocyte–presynapse interactions. In particular, we have previously demonstrated that connexin 43 (Cx43), an astroglial gap junction protein that mediates the extensive and direct communications of astroglial networks [9,10,11], is also involved in sustaining synaptic transmission under physiological conditions [12,13,14,15]. In particular, we showed that the Cx43 conditional knockout in astrocytes results in a reduction in synaptic glutamate release, which, in turn, impairs glutamatergic synaptic activity at CA1 Schaffer collateral hippocampal synapses [15]. More recently, we showed that the specific hemichannel (HC) function of Cx43 contributes to the glutamine supply to presynaptic structures, which occurs in order to support physiological synaptic transmissions and cognitions [13]. While it is clear that astroglial Cx43 is important in maintaining a sustainable pool of glutamate in excitatory synapses, it remains to be determined whether and how this impacts synaptic vesicle dynamics. Here, we focused on the contribution of astroglial Cx43 on presynaptic vesicle recycling by using live imaging of synaptic vesicle release and electrophysiological short-term plasticity assays in acute hippocampal slices. We report that astroglial Cx43 regulates the rate and probability of synaptic vesicle release, without altering the hippocampal neuronal development and expression of key synaptic proteins. These data identify a novel role for Cx43 in maintaining the proper turnover of synaptic vesicles in hippocampal synapses.

## 2. Material and Methods

### 2.1. Mice

All experiments adhered to national and institutional guidelines. Animal experiments were carried out using mice of a wild-type C57BL/6j background (+/+), and mice with a glial conditional deletion of connexin 43 (Cx43) in astrocytes Cx43^fl/fl^:hGFAP-Cre (Cx43−/−). All mice were housed in a 12 h dark/light cycle at 20–21 °C and 45–65% humidity. The Cx43−/− mice were provided by K. Willecke (University of Bonn, Germany) and were characterized prior to the study [16]. Both genders and littermates were used at postnatal days (P) 17–30. All efforts were made to minimize the number of animals used and their suffering.

### 2.2. Acute Brain Slice Preparation

Acute transverse hippocampal slices (300–400 µm) were prepared as previously described [17]. Briefly, mice were sacrificed by cervical dislocation and decapitation. The hippocampi were immediately isolated and sectioned at 4 °C using a vibratome (Leica, Wetzlar, Germany) in an artificial cerebrospinal fluid (ACSF) containing (in mM): 119 NaCl, 2.5 KCl, 2.5 CaCl_2_, 1.3 MgSO_4_, 1 NaH_2_PO_4_, 26.2 NaHCO_3_, and 11 glucose (pH = 7.4). The slices were maintained at room temperature in a storage chamber containing ACSF that was saturated with 95% O_2_ and 5% CO_2_ for at least 1 h prior to experiments.

### 2.3. Electrophysiology and Dye Filling

Acute hippocampal slices were transferred to a submerged recording chamber, which was mounted on an Olympus BX51WI microscope, and which were perfused with ACSF, at a rate of 2 mL/min at room temperature. All electrophysiological recordings were performed in the presence of picrotoxin (100 µM) to block inhibitory transmissions, and a cut was made between the CA3 and CA1 regions to prevent the propagation of epileptiform activity. Field recordings were performed as previously described [18] and in the presence of CPP (10 µM; Tocris Bioscience, Bristol, UK). Field excitatory postsynaptic potentials (fEPSPs) were evoked by Schaffer collaterals stimulation (10–20 µA, 0.1 ms) through an ACSF-filled glass pipette, which was located at a distance of 200 µm from the recording area. Basal activity was measured at 0.1 Hz. Paired-pulse facilitation was recorded by delivering two stimuli at an interval of 40 ms. In some experiments, a Cx43 hemichannel blocker peptide and its scramble version (Gap26/Gap26 scramble; 100 µM; with 5 min pre-incubation) or glutamine (4 mM; with 1–4 h pre-incubation in storage chamber) were included in the ACSF, both before and during the experiments. The recordings were acquired with Axopatch-1D amplifiers (Molecular Devices, San Jose, USA), digitized at 10 kHz, filtered at 2 kHz, as well as stored and analyzed on a computer using pClamp9 and Clampfit10 software (Molecular Devices). Stimulation artifacts were removed in representative traces for illustration. For dye filling experiments, CA1 pyramidal neurons were visually identified and filled under whole-cell configuration via glass pipettes (5–8 MΩ), containing an intracellular solution (in mM): 105 K-gluconate, 30 KCl, 10 HEPES, 10 phosphocreatine, 4 ATP-Mg, 0.3 GTP-Tris, and 0.3 EGTA (pH 7.4, 280 mOsm/L). Sulforhodamine B (1 mg/mL, Sigma-Aldrich, St. Louis, MA, USA) was also included in the intracellular solution in order to label the pyramidal neurons. After dye filling for 20 min, patch pipettes were carefully removed, and slices were fixed for 1 h in 4% PFA at room temperature. In addition, they were directly mounted in Fluoromount-G^TM^ (Thermo Fisher, Waltham, MA, USA) for image acquisition.

### 2.4. Isolation of the Synaptosomal Fractions

The crude synaptosomal fractions from hippocampus were isolated for immunoblotting. First, freshly dissected whole hippocampi were mechanically homogenized using a Potter–Elvehjem homogenizer in an ice-cold homogenization buffer (0.32 M sucrose, 10 mM HEPES, 2 mM EDTA, and protease inhibitors cocktail; 400 µL/hippocampus). The homogenate was then centrifuged at 900× *g* for 15 min at 4 °C. The supernatant was centrifuged at 16,000× *g* for 15 min at 4 °C. The pellet was then washed and resuspended in fresh ice-cold homogenization buffer (300 µL/hippocampus) and centrifuged again at 16,000× *g* for 15 min at 4 °C. The resulting pellet containing synaptosomes was resuspended in a HEPES lysis buffer (50 mM HEPES, 2 mM EDTA, protease inhibitors cocktail; 150 µL/hippocampus). Samples were briefly sonicated to ensure membrane lysis and used for immunoblots. For normalization, the exact protein concentration of each sample was determined using an ionic detergent compatibility reagent (22663, Thermo Fisher). The purity and composition of our hippocampal synaptosomal fractions have been previously verified and reported [13,19,20].

### 2.5. Antibodies and Immunoblotting

The following primary antibodies were used: rabbit anti-Homer1 (1:5000, 160003, Synaptic Systems, Göttingen, Germany); mouse anti-PSD95 (1:5000, 610495, BD Biosciences, Franklin Lakes, NJ, USA); mouse anti-SV2B (1:1000, 119111, Synaptic Systems); rabbit anti-Synapsin 1/2 (1:10,000, 106003, Synaptic Systems); mouse anti-Synaptophysin (1:5000, S5768, Sigma-Aldrich); and mouse anti-VGLUT1 (1:1000, 135511, Synaptic Systems). The following secondary antibodies were used: goat anti-mouse IgG-HRP (1:2500, sc-2005, Santa-Cruz, Dallas, TX, USA) and goat anti-rabbit IgG-HRP (1:2500, sc-2004, Santa-Cruz). All antibodies were commercially available and validated by manufacturers. Western immunoblotting was performed as previously described [18,21]. Equal amounts of protein isolated from the synaptosomal fractions of either +/+ or −/− hippocampus was first separated by electrophoresis in a 10% polyacrylamide gel. This was followed by a transfer of proteins onto nitrocellulose membranes, which were then saturated with a 5% fat-free dried milk in a triphosphate buffer solution and were then incubated overnight at 4 °C with appropriate primary antibodies. On the next day, membranes were treated with peroxidase-conjugated secondary antibodies for 2 h at room temperature and were revealed using a chemiluminescence detection kit (ECL, GE Healthcare, Thermo Fischer) and visualized using a LAS 4000 imaging system and with ImageQuant LAS 4000 software (Fujifilm, Valhalla, NY, USA). The densitometry measurement of protein bands was performed using the *Gel Analysis* function in ImageJ (National Institutes of Health, USA). All readings were normalized to the +/+ bands on the same blot, and a relative expression was plotted.

### 2.6. Two-Photon Live Imaging of Synaptic Vesicle Recycling at Hippocampal Synapses

To quantitatively assess the synaptic vesicle recycling at the hippocampal synapses, we adapted previously published protocols using a FM1-43 dye loading assay in acute brain slices [22,23]. Dye loading and unloading in acute hippocampal slices were controlled by a multi-electrode array (MEA) system and were visualized via two-photon fluorescence live imaging. Briefly, individual 250 µm-thick acute hippocampal slice was first transferred to a planar MEA Petri dish (200-30 indium tin oxide ITO electrodes in 12 × 12 matrix, 30 µm diameter internal reference, 200 µm inter-electrode distance, Multichannel Systems, Reutlinger, Germany), which was then placed on the MEA system (MEA2100-60, Multichannel Systems). It was then mounted below an upright two-photon microscope (MP-2050, Scientifica, Uckfield, UK). Slices were kept under a constant perfusion of oxygenated ACSF at 37 °C, at a rate of 5 mL/min. Before dye loading, the slices were first pre-incubated in FM1-43 (5 µM, Invitrogen, Carlsbad, CA, USA), in the presence of NBQX (10 µM, Tocris Biosciences) for 15 min in order to allow the binding of the dye to the plasma membranes. To load FM1-43 into hippocampal synapses, trains of electrical stimulations (−400 µA, 60 µS; 400 µA, 60 µS) were delivered to the CA1 Schaffer collaterals via 1–2 MEA electrodes at 10 Hz for 2 min; further, the slices were left to rest for 2 additional min. Synaptic activity in the CA1 region was confirmed by an online recording of the relevant electrodes. After dye loading, the excess FM1-43 was washed out by a perfusion of ACSF at 37 °C for 15 min. Successful dye loading was confirmed by a specific punctate fluorescent signal, which was detected using a Mai Tai laser (880 nm, ~0.1 MW, Spectra-Physics, Santa Clara, CA, USA) in the presynaptic terminals with low background non-specific binding. Next, the fluorescence unloading of FM1-43 was monitored using a two-photon live imaging of a region of interest (ROI) in the CA1 region. Image acquisition was performed using 20× water immersion objective with 2× zoom, 12 frames per plane, and 20 planes per stack with z-spacing of 0.5 µm, at a rate of 1 z-stack/15 s. After a baseline recording of 150 s, an unloading stimulus at 10 Hz for 2 min was delivered to the CA1 Schaffer collaterals using the same MEA electrodes that were used for dye loading. MEA was controlled using MC_Rack 4.5.1 software (Multichannel Systems), and image acquisition was performed using SciScan software (Scientifica). Image analysis was performed using ImageJ software (National Institutes of Health). Briefly, the time series stack of individual experiments were generated by concatenating the maximum z-projection of each time frame. Each experiment (156 × 150 µm) was divided into smaller regions of interest (50 × 50 µm) to allow an accurate correction for x-y drift by using the *StackReg* plugin. The change in fluorescence at individual presynaptic puncta was measured using the *Time Series Analyzer* plugin via the use of small circular ROIs of consistent diameter. The resulting fluorescence readings of the individual terminals over time was then exported into Microsoft Excel for further analysis. In total, about 60–90 nerve terminals were analyzed from each experiment. Each individual trace was normalized to the average of 120 s of the baseline level and then plotted as a normalized fluorescence intensity (F/F_0_). The half time (t_1/2_) of dye unloading was determined as the time at which 50% of the baseline fluorescence was unloaded. The mean time constant (τ) of the dye unloading was calculated as the reciprocal of the rate constant. Both t_1/2_ and τ are measures of the rate of synaptic vesicle release from the presynaptic terminals upon activity. The total dye unloading was determined by the relative fluorescence drop between the first minute and last minute of each trace.

### 2.7. Confocal Image Acquisition

Pyramidal neurons filled with sulforhodamine B were imaged in fixed hippocampal slices via a confocal laser-scanning microscope (TCS SP5, Leica) and the image acquisition software LAS X (Leica). The image acquisition was performed using an excitation laser (DPSS 561 nm). For the analysis of neuronal morphology, z-stacks of ~100 μm (with 1 µm z-spacing) were imaged per cell using a 20× objective (NA = 1.25) at 1× zoom. For the analysis of dendritic spine morphology, the z-stacks of 5–10 µm (with 0.2 µm z-spacing) were imaged per dendritic segment by using a 63× objective (NA = 1.4) at 2.5× zoom.

### 2.8. Neuronal Morphology Reconstruction and Analysis

The neuronal morphology was analyzed by using Neurolucida software (MBF Bioscience, Williston, VT, USA). Individual pyramidal neurons were reconstructed using fluorescence images of the entire neuron in 3D, which was achieved by tracing all neuronal branches from the cell soma. The detailed morphometric analysis of neurons were performed subsequently with NeuroExplorer software (MBF Bioscience). The total dendritic length was determined by the sum of either all, basal-only, or apical-only dendrites of each neuron. Polar histograms were generated for each cell to describe the overall direction of dendritic growth, where the length of the dendrites was plotted as a function of discrete angle around the cell soma. The dendritic branching and complexity was measured as the total length of dendrites within the concentric shells around the cell soma. To normalize for potential differences in cell size, both polar histograms and dendritic branching were plotted as fractions of the total length.

### 2.9. Dendritic Spine Analysis

The fluorescence images of the dendritic spines were first deconvolved by using Huygens Essential software (Scientific Volume Imaging B.V., Hilversum, The Netherlands). The analysis of the dendritic spine morphology was performed using the NeuronStudio software package [24]. The following parameters were automatically generated for each spine segment: dendrite length, spine number, spine length, spine head diameter, and spine neck diameter. Spine density was determined as the number of spines per 10 µm of dendrite. Each spine was then categorized as one of the four categories: thin, filopodia, stubby, and mushroom [25]. This was determined mathematically based on the ratios between (1) the spine head diameter and length (HLr) and (2) the spine head and neck diameter (HNr) of the individual spines. Both thin and mushroom spines display a prominent head structure and therefore should have a high HNr (arbitrary threshold set to 1.2). Within this category, the spines with a larger head-to-length ratio (HLr > 0.5) are considered mushroom, whereas those with a smaller ratio are categorized as thin. Other spines with HNr < 1.2 are either stubby (with HLr > 0.5) or filopodia (with HLr < 0.5).

### 2.10. Serial Section Electron Microscopy and Analysis

The hippocampal tissue preparation for ultrastructural analysis, using serial section electron microscopy (EM), was previously described [26]. Briefly, the 3 +/+ and 3 Cx43−/− mice were deeply anesthetized with Narkodorm^®^ (60 mg/kg body weight) and then transcardially perfused with a physiological saline, which was followed by an ice-cold 0.1 M phosphate-buffered (300 mOsm, pH 7.4) fixative, containing 4% paraformaldehyde and 0.1% glutaraldehyde (Polyscience Europe GmbH, Eppelheim, Germany), for 15 min. 100 µm coronal sections of the hippocampus were cut using a vibratome (Leica S1000). After the electron microscopic embedding of the selected hippocampal tissue samples, serial ultrathin sections (~70–80 section within a series; 55 ± 5 nm in thickness, silver to gray interference contrast) were cut using a Leica Ultracut S ultramicrotome; further, they were collected on Pioloform-coated slot copper grids. Prior to transmission electron microscopic examination, the sections were routinely stained with 5% aqueous uranyl acetate for 20 min and a lead citrate for 5 min [27]. The ultrathin sections through the stratum radiatum of the hippocampal CA1 subregion were examined with a Zeiss Libra 120 electron microscope, which was equipped with a Proscan 2K digital camera and the ImageSP software at a magnification of ×8000. Serial EM images (20.46 µm × 20.63 µm) were imported, stacked, and aligned into the software OpenCAR [28]. Subsequently, ~20 randomly chosen synaptic boutons per animal were outlined as contours and 3D reconstructed [29] in order to obtain measurements for the volume of the synaptic boutons and surface areas of postsynaptic densities (PSDs). The values obtained were in line with those recorded in rat CA1 [30]. The measurements of the PSDs were chosen because the electron-dense material of the presynaptic active zones were, unlike those found in the neocortical tissue of other species [31,32,33,34], cannot be unequivocally identified in the synaptic boutons of the CA1 subregion of the hippocampus. However, a perfect overlap in the location and size of the presynaptic active zones and PSDs was, at least, demonstrated in rat layer 5 of the neocortex [35]. Furthermore, the synaptic vesicles and the presynaptic membranes within each bouton that were opposing the PSDs (referred to as presynaptic active zone in this analysis) were marked. This was done in order to analyze the number of synaptic vesicles and the minimal distance between the center of each synaptic vesicle to the corresponding active zone. For the distance measurements, only boutons with a single active zone were chosen for an unambiguous assignment of vesicles to the appropriate active zone. The selected EM images were further processed for final illustrations by using the Adobe Photoshop^TM^ and Illustrator^TM^ software packages.

### 2.11. Statistical Analysis

Data were stored and analyzed using Microsoft Excel (Microsoft, Redmond, WA, USA). The statistical analyses was performed using GraphPad Prism software v7 and v8 (USA). All data are expressed as the mean ± standard error of the mean (SEM), where n represents the number of the independent experiments. The statistical significance was determined by one-sample t-test, unpaired Student’s *t*-test, Mann–Whitney test, one-way ANOVA—or two-way ANOVA—followed by Bonferroni’s multiple comparisons test. The test for normality was performed by using the Shapiro–Wilk normality test. To compare the within-group variance between the experiment groups, F-test was performed.

## 3. Results

### 3.1. Astroglial Cx43 Does Not Alter the Development of Hippocampal Neurons

**Figure 1:**
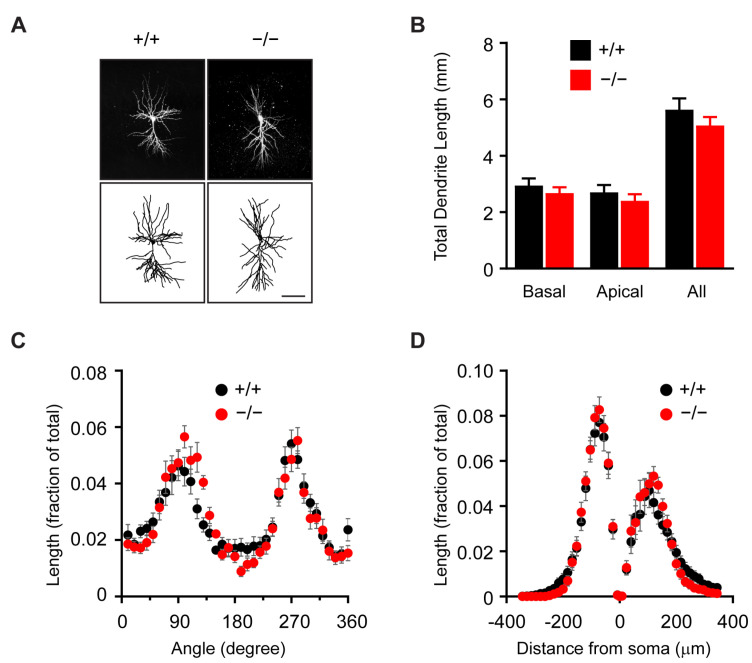
Astroglial Cx43 does not alter neuronal morphology. (**A**) Representative images of a CA1 pyramidal neuron dialyzed with sulforhodamine B via a patch pipette in a wild-type (+/+, left) and a Cx43−/− (−/−, right) hippocampus. The maximum z-projection of the confocal images are shown on the top, and reconstruction of the same neurons are shown at the bottom. (**B**) The plots show the average of the total length of the basal, apical, and all dendrites. (**C**) The fraction of the total dendrites at discrete angles around the cell soma in polar histograms, and (**D**) the fraction of total dendrites intersecting in concentric circles away from the cell soma as shown by Sholl analysis. These analyses show no significant differences in the dendritic organization between pyramidal neurons in +/+ and −/− mice. Mean ± SEM; n = 20 +/+ and 19 −/− cells; and scale bar: 100 µm in (**A**).

**Figure 2:**
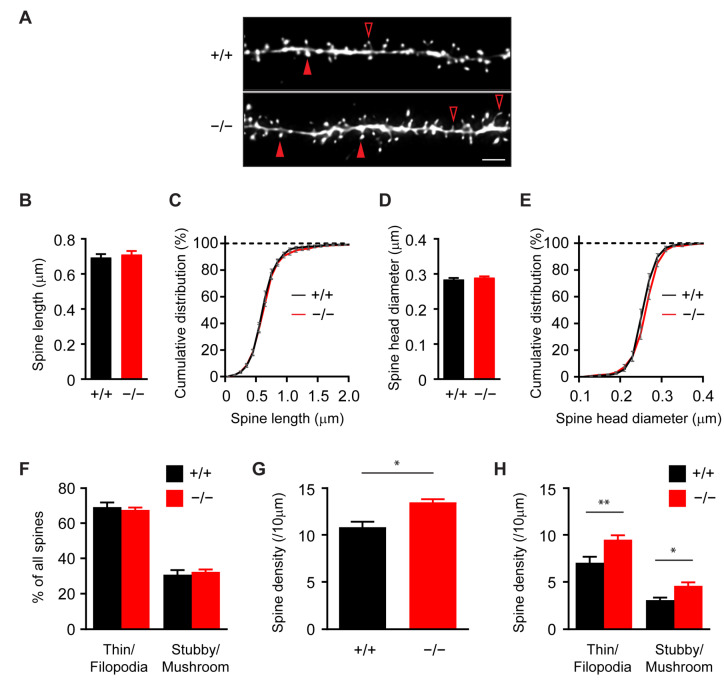
Astroglial Cx43 regulates spine density, but not their properties. (**A**) Representative images of a dendritic segment of a CA1 pyramidal neuron dialyzed with sulforhodamine B via a patch pipette in a wild-type (+/+, top) and a Cx43−/− (−/−, bottom) hippocampus. The maximum z-projection of the confocal images containing dendritic spines are shown. (**B**–**E**) The plots show no significant difference between the averages and cumulative histograms of the spine length (**B**,**C**), nor in the spine head diameter (**D**,**E**) between neurons in +/+ and −/− hippocampi (**F**) The relative proportion of the “Thin/Filopodia” or “Stubby/Mushroom” were not different. (**G**,**H**) Spine density was significantly higher in −/− overall (**G**) and also in both “Thin/Filopodia” and “Stubby/Mushroom” spine categories (**H**). Mean ± SEM; n = 7 +/+ and 6 −/− cells; * *p* < 0.05, ** *p* < 0.01; scale bar: 2 µm in (**A**); and open or filled red arrows denote representative thin/filopodia or stubby/mushroom spines, respectively.

**Figure 3:**
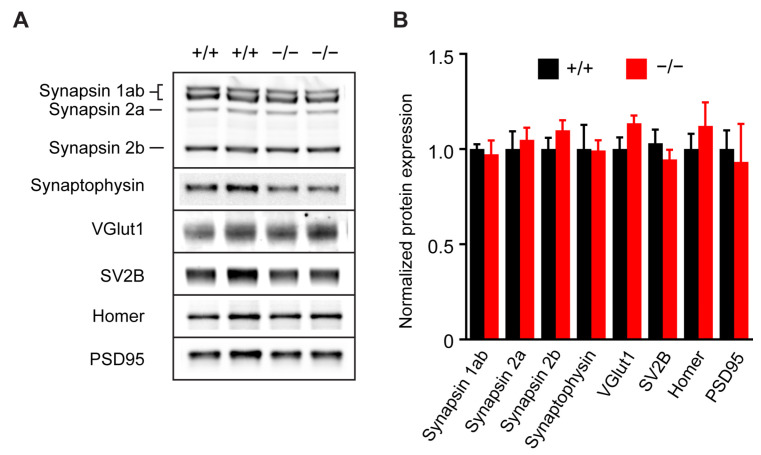
The expression of the key synaptic markers in the hippocampal synaptosomal fractions does not depend on astroglial Cx43 level. (**A**) Representative Western blots show a comparable protein expression of the synaptic markers in synaptosomal fractions, which were isolated from a wild-type (+/+) and a Cx43−/− (−/−) hippocampus. (**B**) The quantification of protein expression normalized to +/+ level for each synaptic marker. Mean ± SEM; n = 5 (+/+) and 4 (−/−).

To assess the role of astroglial Cx43 on synaptic properties, we used a transgenic mouse line with a conditional deletion of Cx43 in the astrocytes (Cx43−/−). Since Cx43 is abundantly and constitutively expressed in the astrocytes early on in the developing brain [36], we first investigated whether Cx43 contributes to the normal development of hippocampal neurons. To this aim, we analyzed the neuronal and dendritic morphologies and expressions of the synaptic markers in hippocampal neurons. We examined the CA1 pyramidal neurons in the acute hippocampal slices, which were prepared from either wild-type (+/+) or Cx43−/− mice, aged 17–21 days old. Individual neurons were identified by morphology and filled with sulforhodamine B (1 mg/mL) for 20 min via a patch pipette. Upon reconstruction of the labeled neurons, we observed no significant morphological difference between the neurons from +/+ and Cx43−/− mice (Figure 1A and Supplementary Figure S1). In particular, we found a comparable total dendritic length (All: 5.64 ± 0.40 mm in +/+, 5.08 ± 0.30 mm in −/−; *p* = 0.2756), which was also similar when considering only either the basal (2.94 ± 0.25 mm in +/+, n = 20; 2.67 ± 0.21 mm in −/−, n = 19; *p* = 0.4255) or apical dendrites (2.70 ± 0.26 mm in +/+, 2.41 ± 0.23 mm in −/−; *p* = 0.4096; unpaired *t*-test; Figure 1B). The overall direction of the dendritic growth measured by the length of dendrites within the discrete angles around the cell body also revealed no difference in the orientation of dendrites between the two conditions (*p* > 0.999; two-way ANOVA; Figure 1C). Finally, dendritic branching and complexity were also unchanged as shown by the Sholl analysis around the somas (*p* > 0.999; two-way ANOVA; Figure 1D). Since astrocytes also have crucial roles in the developmental maturation and functions of dendritic spines [37,38,39], we next investigated the morphology of the CA1 pyramidal neurons at the level of the dendritic spines in the presence or absence of astroglial Cx43. Individual dendritic spines were visible and captured by a high-resolution confocal imaging, which was followed by an image deconvolution of the dendritic segments of sulforhodamine B-filled pyramidal neurons (Figure 2A and Supplementary Figure S2). Considering all spines in general, no difference was detected between +/+ and Cx43−/− mice in terms of spine length (0.69 ± 0.02 μm in +/+, n = 7 neurons; 0.71 ± 0.02 μm in −/−, n = 6 neurons; *p* = 0.6282 in B and *p* = 0.0776 in C) or in terms of the spine head diameter (0.285 ± 0.004 μm in +/+; 0.290 ± 0.003 μm in −/− neurons; *p* = 0.6282 in D and *p* = 0.0611 in E; Mann–Whitney test in B and D; two-way ANOVA in C and E; Figure 2B–E). The dendritic spines were further categorized—into either a thin and filopodia-like immature morphology (“Thin/filopodia”) or a mature stubby and mushroom-like morphology (“Stubby/mushroom”)—which were based on the ratios between their length, head, and neck diameter [25,40]. We found that the proportions of different subtypes of neurons were similar between +/+ and Cx43−/− mice, thereby suggesting an overall similar maturation state (Thin/Filopodia: 69.2 ± 2.6% in +/+; 67.6 ± 1.3% in −/−; Stubby/Mushroom: 30.8 ± 2.6% in +/+; 32.4 ± 1.3 % in −/−; *p* > 0.999; two-way ANOVA; Figure 2F). There was, however, a mild increase in the spine density in Cx43−/− mice (10.83 ± 0.58 in +/+; 13.5 ± 0.32 spines/10 µm in −/−; *p* = 0.035; Mann–Whitney Test; Figure 2G), which was detectable in both of the categories of spines (Thin/Filopodia: 7.04 ± 0.63 in +/+, 9.50 ± 0.47 in −/−, *p* = 0.0021; Stubby/Mushroom: 3.07 ± 0.26 in +/+, 4.58 ± 0.37 spines/10 µm in −/−; *p* = 0.0303; two-way ANOVA; Figure 2H). This observation suggested an over production of all spines without impacting the overall dendritic spine maturation. As we have previously reported that hippocampal excitatory synapse number, labeled by VGlut1 and Homer, was not changed in Cx43−/− mice when compared to +/+ mice [15], the observed increase in dendritic spine density likely does not translate to an increase in the number of functional synapses. However, whether astroglial Cx43 deficiency alters the molecular composition of synapses has not yet been tested. Therefore, we compared the isolated synaptosomal fractions from the hippocampi of either the +/+ or Cx43−/− mice. Immunoblotting revealed that the expression level of key pre- and post- synaptic markers (synapsin 1ab, synapsin 2a, synapsin 2b, synaptophysin, VGlut1, SV2B, homer, and PSD-95) were not altered in the absence of astroglial Cx43 (*p* = 0.7323, *p* = 0.5042, *p* = 0.1598, *p* = 0.8978, *p* = 0.0804, *p* = 0.3951, *p* = 0.3987, *p* = 0.7571, n = 5 +/+ and n = 4 −/−; one-sample unpaired *t*-test; Figure 3). Taken together, these data indicate that astroglial Cx43 does not significantly alter hippocampal neuron morphologies and synaptic molecular compositions.

### 3.2. Astroglial Cx43 Regulates the Distribution of Synaptic Vesicles in Hippocampal Synapses

**Figure 4:**
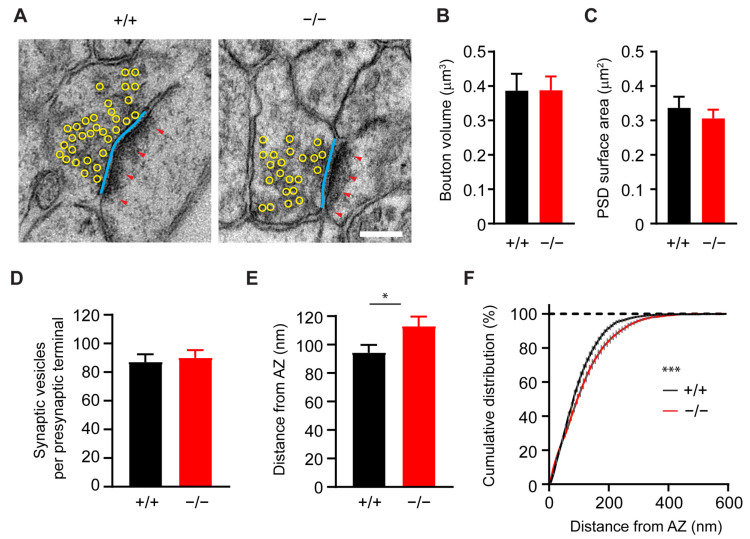
Astroglial Cx43 deficiency disrupts the distribution of synaptic vesicles in presynaptic terminals. (**A**) Representative electron micrographs of hippocampal presynaptic terminals (boutons) in wild-type (+/+, left) and Cx43−/− (−/−, right) mice. Yellow circles outline the synaptic vesicles; blue lines mark active zones; and red arrowheads denote postsynaptic densities (PSD). (**B**–**D**) The plots show no significant difference between the +/+ and −/− mice in terms of the bouton volume (**B**), PSD surface area (**C**), or in the number of synaptic vesicles per presynaptic terminal (**D**). (**E**,**F**) However, the distance of synaptic vesicles from the active zone (AZ) was found to be significantly greater in the −/− mice when compared to the +/+ mice, as can be seen when this is plotted in averages (**E**) and cumulative histograms (**F**). The distance was determined as the minimal distance between the center of each synaptic vesicles to the corresponding presynaptic membrane opposing the PSD. Mean ± SEM; n = 60 (+/+) and 62 (−/−) terminals in (**B**,**C**), as well as the 33 (+/+) and 45 (−/−) terminals in (**D**–**F**). From 3 +/+ and 3 −/− mice; * *p* < 0.05, *** *p* < 0.001; and scale bar: 0.15 µm in (**A**).

We have previously demonstrated the prominent requirement of Cx43 in physiological synaptic transmission and cognition [13,15] in particular via presynaptic regulation. Our data showinged that astroglial Cx43 is not required for the establishment of neuronal morphology in the hippocampus thus points toward a more specific role at the synaptic level. Yet, whether astroglial Cx43 regulates the synaptic vesicle dynamics and recycling, major determinants of presynaptic functions, remains unknown. To investigate this, we first compared the morphology of pre- and post-synaptic compartments at the ultrastructural level. Upon a 3D serial reconstruction of electron microscopic images of the hippocampal synapses in either +/+ or Cx43−/− mice (Figure 4A), we found no significant differences between the presynaptic bouton volume (0.39 ± 0.05 in +/+, n = 60 terminals; 0.39 ± 0.04 in −/−, n = 62 terminals; *p* = 0.6213; Mann–Whitney test; Figure 4B) nor in the surface area of postsynaptic densities (PSD) (0.34 ± 0.03 in +/+; 0.31 ± 0.03 in −/−, *p* = 0.7857; Mann–Whitney test; Figure 4C). Interestingly, we found that while the number of synaptic vesicles per presynaptic terminal did not change upon Cx43 deficiency in astrocytes (86.9 ± 5.5 in +/+, n = 33 terminals; 89.9 ± 5.5 in −/−, n = 45 terminals; *p* = 0.7041; Mann–Whitney test; Figure 4D), they were, on average, distributed further away from the active zone (94.4 ± 5.4 nm in +/+; 112.8 ± 6.8 nm in −/−; *p* = 0.0476 in E and *p* < 0.0001 in F; unpaired *t*-test in E and two-way ANOVA in F; Figure 4E,F). Our observations suggest that astroglial Cx43 deficiency, while not altering pre- and post-synaptic ultrastructure, specifically disrupts synaptic vesicle distribution, which may influence their accessibility during synaptic transmission.

### 3.3. Astroglial Cx43 Controls Synaptic Vesicle Release in Hippocampal Neurons

**Figure 5:**
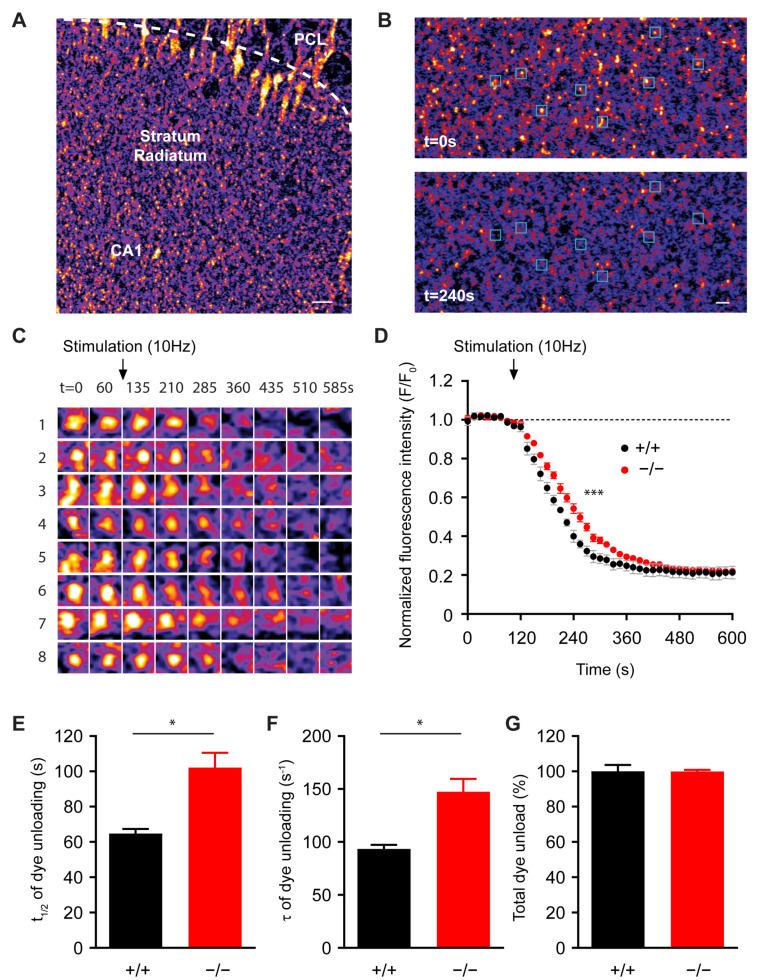
Astroglial Cx43 contributes to synaptic vesicle recycling in hippocampal neurons. (**A**) Representative image of the CA1 stratum radiatum region of a hippocampal slice that is loaded with FM1-43 in presynaptic terminals, as is shown in the fluorescently labeled punctate structures. (**B**) The dye-loaded regions are shown at a higher magnification both before (t = 0) and after (t = 240 s) the dye unloading induced by the 10 Hz stimulation for 2 min. Blue squares mark eight individual synaptic puncta, thereby showing an unloading of FM1-43 dye upon stimulation. (**C**) Time series images of dye unloading are shown for each synaptic puncta marked in (**B**). (**D**) Normalized fluorescence intensity is plotted over time showing the unloading of FM1-43 dye upon stimulation, which is significantly slower in hippocampal slices from Cx43−/− (−/−) mice when compared to wild-type (+/+) (**E**–**G**) The plots showing the average of half time (t_1/2_, **E**) and decay time constant (τ, **F**) of dye unloading, which was found to be significantly higher in −/− when compared to +/+, while, the relative total dye unloaded (**G**) was not different. Mean ± SEM; n = 3 (+/+) and 4 (−/−) independent experiments; * *p* < 0.05; *** *p* < 0.001; and scale bar: 50 µm in (**A**) and 20 µm in (**B**).

**Figure 6:**
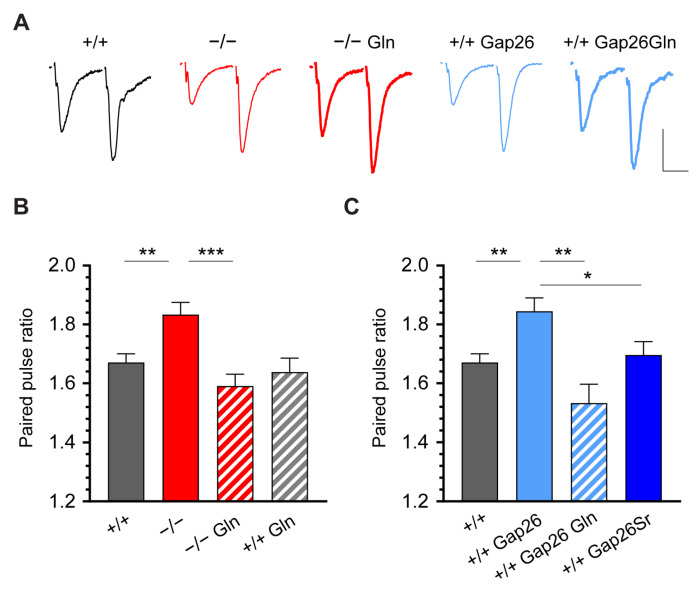
Astroglial Cx43 regulates the release probability in hippocampal neurons. (**A**) Representative recordings showing fEPSPs upon the Schaffer collateral paired-pulse stimulations (10–20 µA, 0.1 ms, 40 ms apart). (**B**) The quantification of the fEPSP amplitude of the second stimulation relative to the first stimulation shows an increase in the paired-pulse ratio in the hippocampal slices from mice that are deficient in astroglial Cx43. These were then rescued by an exogenous application of glutamine (4 mM, −/− Gln, 1–4 h). Glutamine alone did not alter the paired pulse ratio (+/+ Gln). (**C**) This effect was mimicked by Gap26 in the +/+ slices (+/+ Gap26), but not by a scrambled version of Gap26 (+/+ Gap26Src) and rescued by exogenous glutamine (+/+ Gap26 Gln). Mean ± SEM; n = 19 (+/+), 16 (−/−), 9 (−/− Gln), 8 (+/+ Gln), 17 (+/+ Gap26), 5 (+/+ Gap26 Gln), and 12 (+/+ Gap26Src) independent experiments; * *p* < 0.05, ** *p* < 0.01, *** *p* < 0.001; and scale bars: 0.2 mM, 20 ms in (**A**).

A disruption of synaptic vesicle arrangements in the presynaptic terminals suggest a potential impairment of synaptic vesicle release in the absence of astroglial Cx43. Thus, we next quantitatively assessed the dynamics of synaptic vesicle recycling in acute hippocampal slices by combining two-photon live imaging and multi-electrode array (MEA) stimulations. Acute hippocampal slices were first placed on a MEA system and loaded with FM1-43 (5 µM), which is a styryl dye that reversibly binds to plasma membrane and is taken up by nerve terminals through endocytosis, upon synaptic activity [41]. FM1-43 was loaded using electrical stimulation at 10 Hz for 2 min delivered to the CA1 Schaffer collaterals by using MEA electrodes. This resulted in the live labeling of presynaptic nerve terminals as fluorescence puncta throughout the slices (Figure 5A). A second train of electrical stimulation at 10 Hz for 2 min delivered using the same MEA electrodes triggered synaptic vesicle fusion to the presynaptic membrane during exocytosis. The drop of fluorescence, as a result of vesicle fusion, was then monitored at each nerve terminal by using two-photon live imaging (Figure 5B,C). Upon the quantification of individual nerve terminals, we observed a significantly slower synaptic vesicle release rate in mice that were deficient in astroglial Cx43 when compared to the +/+ mice, as was indicated by a prolonged fluorescence decay (*p* < 0.001; n = 3 +/+ and n = 4 −/−; two-way ANOVA; Figure 5D) with a longer half-time (t_1/2_; 64.7 ± 2.6 s in +/+; 102.2 ± 8.4 s in −/−; *p* = 0.0144; unpaired *t*-test; Figure 5E) and decay time constant (τ; 93.4 ± 3.8 s in +/+; 147.4 ± 12.1 s in −/−; *p* = 0.0144; unpaired *t*-test; Figure 5F). The relative amount of unloaded dye was not different, thereby suggesting that, while the releasable vesicle pool size was comparable, it took longer for hippocampal nerve terminals in Cx43−/− mice to deplete this pool (99.9 ± 0.9% in −/− compared to 100% in +/+; *p* = 0.9476; one-sample *t*-test; Figure 5G). One mechanism to account for a slower synaptic vesicle release at hippocampal synapses in the absence of astroglial Cx43 may consist of a general impairment of synaptic vesicle release probability. To test this hypothesis, we performed field recordings in hippocampal slices upon paired-pulse stimulations of Schaffer collaterals (two stimulus delivered 40 ms apart). Paired-pulse facilitation (PPF) in the +/+ slices was observed as a form of a typical short-term plasticity response in the excitatory synapses (Figure 6A). However, the hippocampal slices obtained from Cx43−/− mice showed a significant increase in a paired-pulse ratio (1.67 ± 0.03 in +/+ to 1.83 ± 0.04 in −/−; *p* = 0.0071; Figure 6A,B), thus suggesting a lower release probability at these synapses, which corroborates our findings from the FM1-43 assays. As the hemichannel (HC) function of Cx43 was found to support the physiological synaptic transmission by supplying presynaptic elements with glutamine [13], we further investigated whether the role of Cx43 in release probability, was mediated by a similar mechanism here. To do this, we measured PPF of the +/+ slices in the presence of Gap26 (100 µM), which is a specific blocker of Cx43 HC activity that was used and verified in our previous studies [13,14]. We observed a similar impairment in terms of the release probability using Gap26, as observed in the Cx43−/− mice, while the scrambled version of the peptide (Gap26Sr; 100 µM) had no effect (1.85 ± 0.05 in +/+ Gap26, *p* = 0.004 with +/+; 1.70 ± 0.04 in +/+ Gap26Sr, *p* = 0.0468 with +/+ Gap26; one-way ANOVA; Figure 6A,C). Remarkably, the reduced release probability elicited by astroglial Cx43 deficiency, or by Gap26, was rescued by an exogenous supply of glutamine (4 mM for 1–4 h; 1.59 ± 0.04 in −/− Gln, *p* = 0.0008 with −/−; 1.53 ± 0.06 in +/+ Gap26 Gln, *p* = 0.0005 with +/+ Gap26; one-way ANOVA; Figure 6A–C). We chose an incubation period of under 4 h to prevent the enhancement of basal synaptic transmissions, which was previously reported for prolonged incubations [42] and verified this by showing no effect 4 mM Gln on the PPF in the +/+ slices (1.64 ± 0.05 in +/+ Gln, *p* = 0.5412 with +/+; Mann–Whitney Test; Figure 6B). We have also previously confirmed that this treatment does not affect physiological synaptic transmission evoked by 10 Hz stimulation in the +/+ hippocampal slices [13]. We tested a range of incubation duration between 1 and 4 h, but found no time-dependent difference (comparable coefficient of variation between the experimental groups with or without Gln; *p* = 0.8226 between +/+ and +/+ Gln; *p* = 0.3745 between −/− and −/− Gln; *p* = 0.6478 between +/+ Gap26 and +/+ Gap26 Gln). The experiments obtained over 1–4 h incubation were therefore pooled for analysis. Thus, our findings indicate that the synaptic vesicle release probability also relies on glutamine supply to neurons via a Cx43 HC-dependent mechanism. Taken together, using both live imaging and electrophysiology, we found that astroglial Cx43 modulates synaptic vesicle release from hippocampal synapses via its HC function supplying glutamine to presynaptic compartments.

## 4. Discussion

Our data show that while the Cx43 in astrocytes is not required for the proper establishment of neuronal structural properties and the expression of key synaptic proteins, it has a significant role in sustaining presynaptic efficacy in hippocampal neurons. In particular, we report an altered distribution of synaptic vesicles, as well as a reduced rate and probability of synaptic vesicle release at the hippocampal synapses, specifically in the absence of astroglial Cx43. Vesicular glutamate concentration is a potential source of variations in quantal size [43]. While release probability is not traditionally thought to be influenced by quantal size [44], it has recently been shown in hippocampal neurons to be modulated by synaptic vesicle acidification and glutamate transporter expression [45,46], which are known determinants of synaptic quantal size [47]. Additionally, cytosolic factors, such as neurotransmitter concentrations, also control synaptic vesicle quantal size [47]. In the calyx of Held synapses, an increase in quantal size and mEPSC amplitudes was observed when presynaptic terminals were supplied with excess glutamate [48]. These evidence, together with our previous reports that synaptic glutamate concentration as well as mEPSC amplitude decreased in the absence of astroglial Cx43 [15], further explains the impairment in synaptic vesicle release probability that was observed in this study. Our observation that synaptic vesicles were further away from the active zone in the absence of astroglial Cx43 correlates with the observed impairment of their release and is in line with previous reports showing that synaptic vesicles distribution at the active zone correlates with the release probability in hippocampal excitatory synapses [49]. Moreover, astrocytes are also known to recycle synaptic glutamate by supplying neurons with glutamine during the process of the glutamate–glutamine cycle [8]. In fact, astrocytes are not only the major source of synaptic glutamate, but they also have significant roles in maintaining glutamate homeostasis in the CNS [50]. We showed that under physiological conditions, hippocampal synaptic transmission does indeed rely upon astroglial glutamine supply in a Cx43 HC-dependent manner [13]. An imbalance in this cycle would have a direct impact on the pool of glutamate for neurons to continue neurotransmission. Remarkably, blocking Cx43 HC activity alone is sufficient to reduce synaptic vesicle release probability to the same extent as Cx43 deficiency in astrocytes. This suggests that an acute activation of Cx43 HC is required to sustain synaptic vesicle release. Further to this, the rescue of the slowed synaptic vesicle release by exogenous glutamine supply strongly suggests a mechanism that is dependent on the maintenance of synaptic vesicle content. These findings, therefore, extend our understanding of the role of astroglial Cx43 in the regulation of physiological synaptic transmission via the replenishment of presynaptic glutamate and the modulation of synaptic vesicle release probability. Of note is the specificity of Cx43 knockout when it is driven by an astroglial-specific *hGFAP* promoter, which has been previously validated to be specific to astrocytes, but not in microglia or oligodendrocytes [16]. Given that the expression of Cx43 is not detectable in oligodendrocytes, and only in negligible level in resting microglia [51], our observations reflect a specific Cx43 function in astrocytes. While focusing on the interaction between astrocytes and neuronal synapses in this study, any indirect effect on synaptic properties via other cell types as a result of manipulating astroglial Cx43 function has not been assessed. The current study continues our previous work on the specific role of astroglial Cx43 on excitatory synapses and synaptic glutamate [15] and presynaptic glutamine [13] dynamics. However, its potential effect on inhibitory synaptic transmission and network was not tested. Nevertheless, since electrophysiological recordings were performed in the presence of picrotoxin, an action of inhibitory network through GABA_A_ receptors does not contribute to the observed effect. When examining neuronal morphologies in mice with astroglial Cx43 deficiency, we only observed an overall mild increase in dendritic spine density. As synaptic transmission is a crucial factor sustaining and stabilizing dendritic spines in the developing hippocampus [52], it is plausible that the reduced synaptic transmission in mice with astroglial Cx43 deficiency alters dendritic spine dynamics. Furthermore, our data are also consistent with an increase in spine density that was documented as a result of a reduced synaptic transmission in the hippocampal slices from both juvenile and adult mice, but not young mice, suggesting an age-dependent homeostatic structural plasticity [53]. On the contrary, a loss of dendritic spines was observed during excessive stimulation of synapses, further highlighting the dynamic plasticity of dendritic spines both in vivo [54] and in situ [55]. Based on this, we postulate that the mild increase in spine density that we observe in this study could be a compensatory response to a chronic inhibition of synaptic transmission [56]. Since we observed that the increase in spine density does not have any differential impact on immature and mature spines, and the total excitatory synapse numbers are not changed in these mice [15], this outcome is unlikely to impact spine maturation and synapse formation. Nevertheless, our observation highlights a potentially significant long-term effect of astroglial Cx43 deficiency on the production and elimination of dendritic spines. In summary, our findings, focusing on synaptic vesicle release by hippocampal neurons, provide important further insights into the role of Cx43 in physiological synaptic transmission.

## Data Availability

All relevant data are included in the paper and/or its Supplementary Materials.

## Supplementary Materials

The following supporting information can be downloaded at: https://www.mdpi.com/article/10.3390/cells12081133/s1, Figure S1. Dendritic morphology of CA1 pyramidal neurons is not changed in mice that are deficient in astroglial Cx43. Figure S2. Dendritic spine properties of CA1 pyramidal neurons are not changed in the absence of astroglial Cx43. Figure S3. The original Western blots shown in Figure 3.
