# Supplementary material for: Astroglial Connexin 43 Regulates Synaptic Vesicle Release at Hippocampal Synapses
Source: Cells. 2023 Apr 11;12(8):1133. doi: 10.3390/cells12081133 (PMC10136730; doi:10.3390/cells12081133)
Supplement: Supplementary file 1 [file cells-12-01133-s001.zip › cells-2257639-supplementary.pdf]

Supplementary Materials

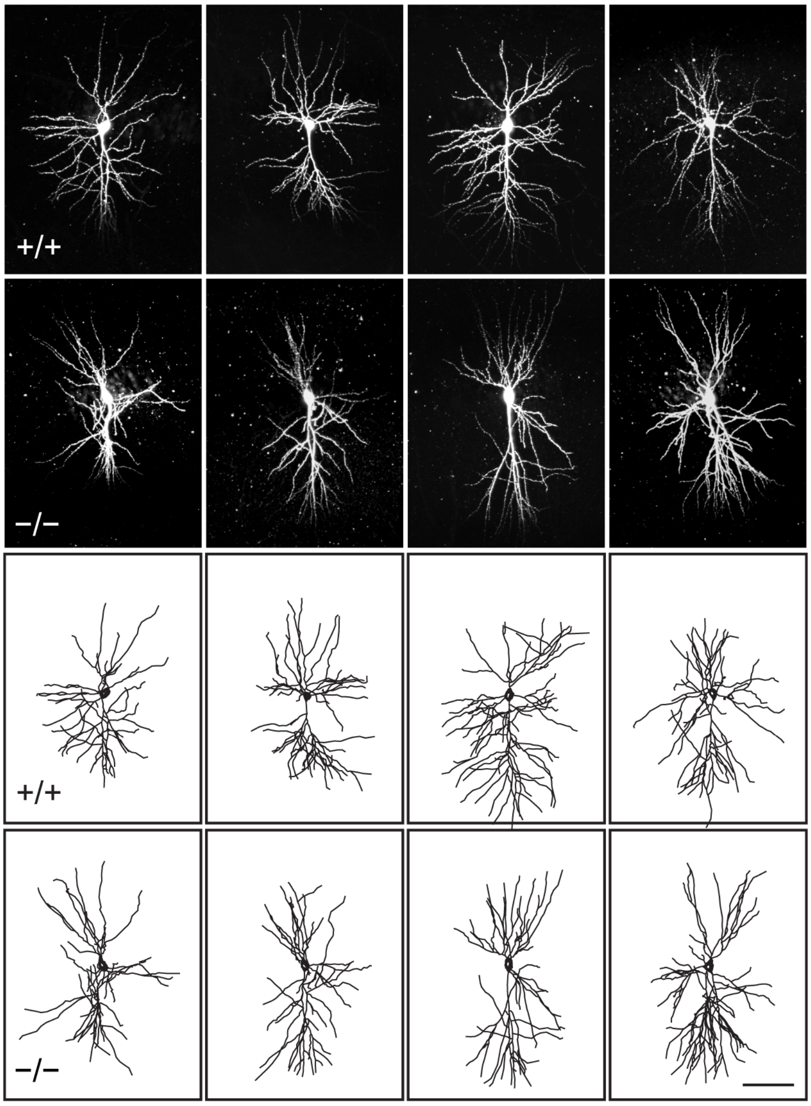

Figure S1. Dendritic morphology of CA1 pyramidal neurons is not changed in mice deficient for astroglial Cx43. Representative images of four hippocampal CA1 pyramidal neurons filled with sulforhodamine B via a patch pipette in wildtype (+/+, top) and Cx43 −/− (−/−, second from top) mice. Maximum z-projection of confocal images are shown on the top two panels and reconstruction of the same neurons at the bottom two panels. Scale bar: 100 μm.

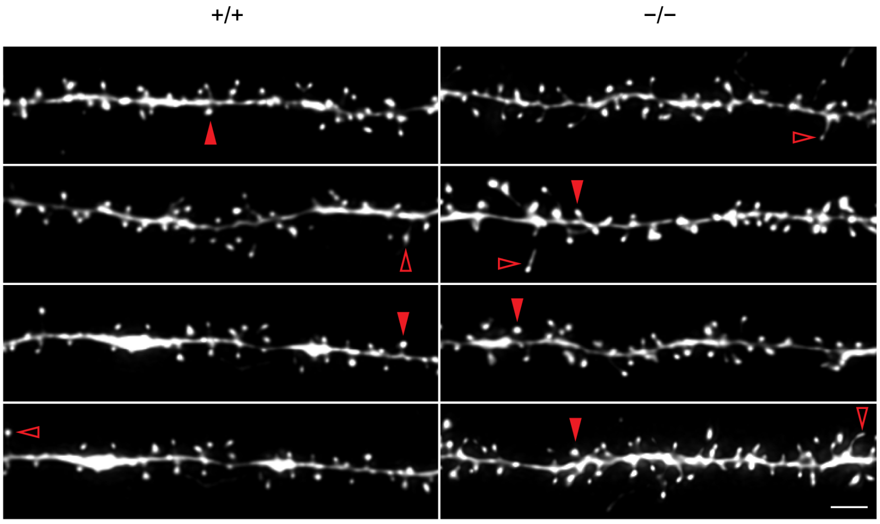

Figure S2. Dendritic spine properties of CA1 pyramidal neurons are unaltered in the absence of astroglial Cx43. Representative images of four dendritic segments of hippocampal CA1 pyramidal neurons filled with sulforhodamine B via a patch pipette in wild type (+/+, left) and Cx43 −/− (−/−, right) mice. Maximum z-projection of confocal images containing dendritic spines are shown. Scale bar: 2 µm. Open or filled red arrows denote representative thin/filopodia or stubby/mushroom spines, respectively.

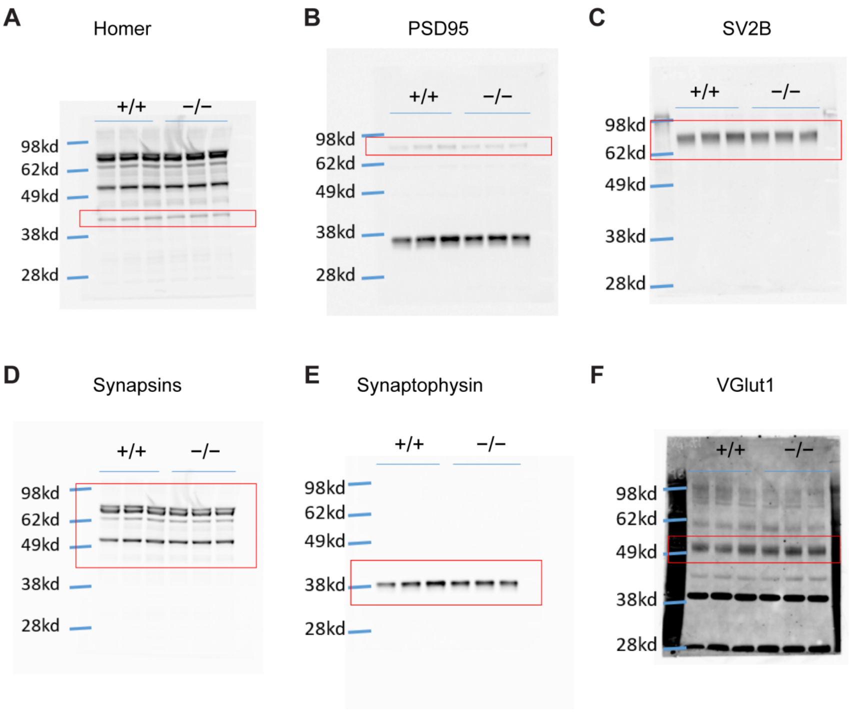

Figure S3. Original western blots shown in Figure 3. Original uncropped western blots used for protein quantification in Figure 3 are shown for **(A)** Homer, **(B)** PSD96, **(C)** SV2B, **(D)** Synapsins, **(E)** Synaptophysin, and **(F)** VGlut1.
